# Investigating and modeling positron emission tomography factors associated with large cell transformation from low‐grade lymphomas

**DOI:** 10.1002/jha2.615

**Published:** 2022-11-25

**Authors:** Jean‐Pierre Obeid, Susan M. Hiniker, Joseph Schroers‐Martin, H. Henry Guo, Hyunsoo Joshua No, Everett J. Moding, Ranjana H. Advani, Ash A. Alizadeh, Richard T. Hoppe, Michael S. Binkley

**Affiliations:** ^1^ Department of Radiation Oncology Stanford University School of Medicine Stanford California USA; ^2^ Department of Medicine Division of Oncology, Stanford University School of Medicine Stanford California USA; ^3^ Department of Radiology Stanford University School of Medicine Stanford California USA

**Keywords:** lymphomas, mathematical modeling, pet

## Abstract

Low‐grade lymphomas have a 1%–3% annual risk of transformation to a high‐grade histology, and prognostic factors remain undefined. We set to investigate the role of positron emission tomography (PET) metrics in identification of transformation in a retrospective case‐control series of patients matched by histology and follow‐up time. We measured PET parameters including maximum standard uptake value (SUV‐max) and total lesion glycolysis (TLG), and developed a PET feature and lactate dehydrogenase (LDH)‐based model to identify transformation status within discovery and validation cohorts. For our discovery cohort, we identified 53 patients with transformation and 53 controls with a similar distribution of follicular lymphoma (FL). Time to transformation and control follow‐up time was similar. We observed a significant incremental increase in SUV‐max and TLG between control, pretransformation and post‐transformation groups (*P* < 0.05). By multivariable analysis, we identified a significant interaction between SUV‐max and TLG such that SUV‐max had highest significance for low volume cases (*P* = 0.04). We developed a scoring model incorporating SUV‐max, TLG, and serum LDH with improved identification of transformation (area under the curve [AUC] = 0.91). Our model performed similarly for our validation cohort of 23 patients (AUC = 0.90). With external and prospective validation, our scoring model may provide a specific and noninvasive tool for risk stratification for patients with low‐grade lymphoma.

## 1 INTRODUCTION

Relapses of indolent lymphomas typically remain low‐grade, but there is an estimated 1%–3% annual risk of transformation to an aggressive histology, most commonly, large B‐cell lymphoma [1]. For follicular lymphoma (FL), patients with grade 3 histology, advanced stage disease, high FL international prognostic index, and elevated serum beta‐2 microglobulin have been reported as having higher risk of transformation [2, 3, 4, 5]. For nodular lymphocyte‐predominant Hodgkin lymphoma (NLPHL), prior studies have reported an association between splenic disease, advanced stage, increasing number of involved sites, and variant immunoarchitectural pattern with risk of transformation [6, 7]. However, prognostic factors at the time of initial lymphoma diagnosis and at time of transformation remain incompletely defined. Identification of such factors associated with higher risk of transformation may be clinically useful in individualizing treatment intensity. For example, the TROG 9903‐randomized clinical trial reported numerically fewer transformations for patients who received combined modality therapy versus those that received radiotherapy alone, suggesting intensity of therapy may impact incidence of transformation [8].

Given the high sensitivity of ^18^F‐fluorodeoxyglucose (FDG) positron emission tomography (PET) in detecting the extent of lymphoma involvement [9], there has been increasing interest in the possibility of using PET metrics, such as the standardized uptake value (SUV) maximum (SUV‐max), as imaging‐based biomarkers for identification of patients with more aggressive histology disease [10, 11, 12]. However, studies incorporating clinical factors, total lesion glycolysis (TLG), and metabolic tumor volume (MTV) in combination with SUV‐max have not been studied as robustly.

We sought to investigate whether PET metrics as well as clinical factors may have utility in identification of histologic transformation to aggressive large cell lymphoma in patients previously diagnosed with low‐grade lymphomas using a case‐control design.

## 2 METHODS

### 2.1 Patients

We performed a retrospective case‐control study of patients seen at the Stanford Cancer Center from 2003 through 2020 with institutional board review (IRB) approval. Our inclusion criteria for cases included a diagnosis of an indolent lymphoma, including but not limited to FL (grade I‐3A), marginal zone lymphoma, mantle cell lymphoma, and NLPHL which preceded a diagnosis of large cell transformation or a clinical transformation consisting of aggressive disease behavior with associated intensification in management [6]. Patients were included if their date of transformation was within the study date range. Inclusion criteria for the control group included a diagnosis of a low‐grade lymphoma without large cell or clinical transformation. We used an institutional cohort discovery tool called STRIDE [13] to identify patients. To identify transformed cases, diagnosis search keywords included “indolent lymphoma,” “FL,” “marginal zone lymphoma,” and “diffuse large B‐cell lymphoma.” Patients were randomly selected after our search with manual review performed to confirm presence of an indolent lymphoma prior to a verified large‐cell transformation. To identify control patients, diagnosis search keywords included “indolent lymphoma,” “FL,” “marginal zone lymphoma,” and the explicit lack of “diffuse large B‐cell lymphoma” with manual verification.

We extracted clinical demographic information including date of birth, gender, living status, and last date of follow‐up or death. Pathology was reviewed at our institution at the time of diagnosis. We reviewed clinical notes, imaging, pathology reports to record the date of diagnosis, biopsy site, histologic diagnosis, stage, Ann Arbor sites of involvement, lactate dehydrogenase (LDH) elevation status at diagnosis and at transformation (relative to laboratory reference), and management with surgery, systemic therapy or radiation.

### 2.2 Imaging

^18^F‐FDG PET was required either at the time of transformation, prior to transformation or both. When obtained prior to transformation, PET was either at time of initial diagnosis or time of relapse with a pathologically confirmed low‐grade lymphoma. All PET acquisitions in this study were obtained with corresponding computed tomography for attenuation correction and anatomic mapping. Although imaging protocols varied over the course of our study, in general after confirmation of a serum glucose <180 mg/dl, patients received 12–18 mCi ^18^F‐FDG 45–60 min prior to their scan. Prior to 2013, PET data were reconstructed with an ordered set expectation maximization algorithm and afterward using a time‐of‐flight point‐spread function.

We extracted PET characteristics from regions of interest (ROIs) by contouring the PET‐avid sites in each patient's study. For this we used the MIM software (Version 7.1.2, MIM Software Inc., Cleveland, OH) PET Edge gradient‐based tumor‐segmentation tool to define the MTV [14, 15]. We also performed site segmentation using 41% of the SUV‐max given its prevalence in the literature to ensure consistency among delineation methods [16, 17]. Post‐contouring analysis involved quantifying the number of region of interest (ROI) volumes corresponding to distinct nodal or extranodal lymphoma involvement, the global SUV‐max, the TLG and the MTV among all summed ROIs. TLG was obtained by multiplying the MTV by the average SUV in the volume.

### 2.3 Statistical analysis and predictive modeling

We measured follow‐up time from the date of diagnosis to the date of last follow up visit or death date. Follow‐up time was measured using the reverse Kaplan–Meier method. Overall survival (OS) was measured using the Kaplan–Meier method with differences compared using the log‐rank test. Time to transformation was defined as the time from diagnosis of low‐grade lymphoma to either pathologic confirmation of large‐cell transformation or clinical diagnosis of transformation. We compared baseline characteristics of the transformation group and the control group using Student's *t*‐test and analysis of variance for continuous variables and Fisher's exact test for categorical data. We performed univariable and multivariable (MVA) logistic regression and receiver operator characteristic (ROC) curves to assess association between patient factors and transformation events. We also performed univariable and MVA competing risk regression analyses adjusting for the competing risk of death including factors with *P* < 0.05 on univariable analysis for inclusion in the multivariable analysis.

For the discovery cohort, we matched cases to controls at a 1:1 ratio using two criteria. For the first matching condition, we sought to equalize the proportion of FL versus non‐FL cases present in each cohort as that was the most common histology identified by our cohort discovery. To minimize the selection bias that may be introduced by selecting cases by histology, we also performed a stratified analysis of FL only cases. For the second condition, we matched the control patients based on follow‐up time mean and standard deviation to the time to transformation for the cases. This was done to reduce the risk of missing future transformations in the identified control group. Following successful matching, clinical comparability between the groups was verified via Follicular Lymphoma International Prognostic Index (FLIPI) score within the FL subgroups.

We evaluated the performance of several predictive models including logistic regression models with variables included as linear terms with or without interaction terms as well as interaction terms alone if variables were nested (such as PET metrics, [18]). After visualization of data and evaluating univariable influences, we generated a mathematical model to differentiate between transformed and control low‐grade cases based on PET metrics. The model outputs a score, which can be calculated by taking the product of SUV‐max with the base‐10 logarithm of (1 + TLG) and with an LDH scaling factor. The scaling factor takes on an integer value of 1 if LDH at diagnosis is not elevated above laboratory refence and 2 if LDH at diagnosis is elevated.

$$\mathrm{Score}=SUV_{\max}*\log_{10}\left(1+TLG\right)*K_{\mathrm{LDH}}$$

For all analyses, we used a predefined two‐tailed level of statistical significance (SS, alpha) chosen to be <0.05. All computations, analyses and figures were generated using Python software (Version 3.8.6, Python Software Foundation, Wilmington, DE) and R software (version 3.6, Vienna, Austria).

### 2.4 Validation cohort

For internal validation, we identified a small independent cohort of patients with initial low‐grade lymphoma diagnosis with a subset experiencing low‐grade to high‐grade transformation during follow‐up. We identified this cohort using our same database and search criteria as used for the discovery cohort. We did not perform a 1:1 match for the validation cohort of transformation and control cases as we sought to measure model performance in a less stringently selected cohort and due to difficulty identifying additional patients meeting our inclusion criteria. All patients in the validation cohort had a PET scan at either time of low‐grade lymphoma diagnosis or at time of transformation. Similar to our discovery cohort, we extracted clinical and radiological PET features. We employed our PET‐metric and LDH‐based scoring model to this independent validation cohort to measure its performance.

## 3 RESULTS

### 3.1 Patients, management, and follow up

For our discovery cohort, a total of 106 patients (53 cases and 53 controls) were identified meeting our inclusion criteria. Baseline characteristics including age, gender, and LDH elevation at diagnosis of low‐grade lymphoma were similar between cases and controls and were not significantly different as summarized in Table 1 (*P* > 0.05). There was a nonsignificantly higher percentage of patients with advanced stage at initial diagnosis of low‐grade lymphoma that developed transformation relative to the control cohort (*P* = 0.14). FL was the most common histology identified by our cohort discovery tool with successful matching to ensure a similar percentage of FL achieved between cases (79%) and controls (77%, *P* = 1.0). Among the 21% of non‐FL diagnoses in the cases, histologies included marginal zone lymphoma, Waldenstrom macroglobulinemia, NLPHL, mantle cell lymphoma, and primary cutaneous follicle center lymphoma. For the 23% of non‐FL diagnoses in the controls, histologies included marginal zone and NLPHL.

**TABLE 1:** Summary of characteristics of patients who experienced transformation to large cell lymphomas (cases) and those with low‐grade lymphomas without transformation (controls)

Variables	Transformation Group, *n* (%)	Control group, *n* (%)	* P* ‐Value
*N*	53 (100%)	53 (100%)	
Gender	Male: 31 (58%) Female: 22 (42%)	Male: 25 (47%) Female: 28 (53%)	0.33
Age – LG (years)	Median: 57, (range, 34 – 73)	Median: 60 (range, 29–80)	0.31
Histology – LG	FL: 42 (79%) Non‐FL: 11 (21%)	FL: 41 (77%) Non‐FL: 12 (23%)	1.0
Stage – LG	I: 8 (16%) II: 6 (12%) III: 19 (37%) IV: 18 (35%)	I: 15 (28%) II: 13 (25%) III: 9 (17%) IV: 16 (30%)	0.14
FLIPI score – LG (FL)	0: 1 (3%) 1: 15 (45%) 2: 9 (28%) 3: 7 (21%) 4: 0 (0%) 5: 1 (3%)	0: 5 (14%) 1: 8 (23%) 2: 15 (43%) 3: 7 (20%) 4: 0 (0%) 5: 0 (0%)	0.12
LDH elevated/available – LG	2/31 (6%)	6/44 (14%)	0.46
Management – LG	Observation: 9 (17%) Definitive resection: 2 (4%) Systemic: 33 (62%) Definitive radiation: 8 (15%) Palliative radiation: 1 (2%)	Observation: 6 (11%) Definitive resection: 2 (4%) Systemic: 31 (58%) Definitive radiation: 14 (26%) Palliative radiation: 0 (0%)	0.63
Time‐to‐transform (months)	Mean: 100.9 (range, 4.9–306.4) Median: 102.2		
Follow‐up time (months)	Mean: 158.2 (range, 12.8–350.0) Median: 152.1	Mean: 96.0 (range, 3.9–305.3) Median: 88.1	< 0.01
Age – HG (years)	Median: 66, (range, 41–83)		
Histology – HG	DLBCL: 47 (89%) HGBL: 3 (6%) FL: 2 (4%) Histiocytic sarcoma: 1 (2%)		
Stage – HG	I: 7 (13%) II: 6 (11%) III: 10 (19%) IV: 30 (57%)		
LDH elevated/available – HG	28/47 (60%)		
Management – HG	Definitive resection: 0 (0%) Systemic: 53 (100%) Definitive radiation: 0 (0%) Consolidation radiation: 11 (21%) Palliative radiation: 7 (13%)		

Abbreviations: DLBCL, diffuse large B‐cell lymphoma; FL, follicular lymphoma; FLIPI, Follicular Lymphoma International Prognostic Index; HG, high‐grade; HGBL, high‐grade B‐cell lymphoma; LG, low‐grade.

As summarized in Table 1, management prior to transformation for the cases included definitive resection without known residual lymphoma for 2 (4%), rituximab monotherapy for 13 (25%), chemotherapy with or without rituximab for 16 (30%), a vaccine trial for 4 (8%), definitive radiotherapy in 8 (15%), and palliative radiotherapy for 1 (2%). Observation, consisting of no treatment prior to transformation, was selected for nine (17%) patients. Median follow‐up time from initial diagnosis was 152.1 months (range: 12.8–350.0). Median time to transformation was 102.2 months (mean: 100.9, range: 4.9–306.4). Of the 42 cases with initial diagnosis of FL, FLIPI score was present in 33 (79%) patients. FLIPI distribution was one (3%) patient score 0, 15 (45%) score 1, 9 (28%) score 2, 7 (21%) score 3, 0 (0%) score 4, and 1 (3%) score 5 with a mean of 1.79. Following transformation, high‐grade histology was diffuse large B‐cell lymphoma (DLBCL) in 47 (89%) patients, high‐grade B lymphoma in 3 (6%), FL in 2 (Grade 3B, 4%), and histiocytic sarcoma in 1 (2%). Post‐transformation stage distributions were 7 (13%) patients in stage I, 6 (11%) in stage 2, 10 (19%) in stage III and 30 (57%) in stage IV. LDH at the time of transformation was available in 47 patients, among whom it was elevated in 28 (60%) patients. Treatment post‐transformation was systemic therapy for all (100%) patients with 11 (21%) receiving consolidative radiotherapy and 7 (13%) receiving palliative radiotherapy (Table 1). Combining all low‐grade (pretransformed and control) patients, LDH elevation in eight of 75 such patients were statistically significantly different from LDH elevation in 28 of 47 post‐transformation cases by Fisher's test (*P* < 0.001).

**FIGURE 1:**
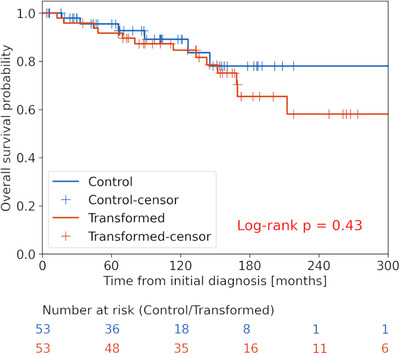
**Overall survival (OS) for patients who experienced transformation to large cell lymphoma versus those who did not**. Kaplan–Meier curves demonstrate no significant difference in OS for patients who experienced transformation (red) versus those who did not (blue, *P* = 0.43).

Initial management for the controls included definitive resection for 2 (4%), rituximab monotherapy for 13 (9%), chemotherapy with or without rituximab for 16 (49%), a vaccine trial for 2 (4%), and definitive radiotherapy for 14 (26%). Observation without any treatment was employed for 6 (11%) patients. Median follow‐up time after diagnosis was 88.1 months (mean: 96.0, range: 3.9–305.3). Of the 41 controls with initial diagnosis of FL, FLIPI score was present in 35 (85%) patients. FLIPI distribution was five (14%) patients score 0, 8 (23%) score 1, 15 (43%) score 2, 7 (20%) score 3, 0 (0%) score 4, and 0 (0%) score 5 with a mean of 1.69. Importantly, there was no difference between the control follow‐up time distribution and the time to transformation distribution suggesting our matching was successful with sufficient follow‐up time of controls to observe for transformation events (*P* = 0.715). Similarly, there was no difference between the distributions of the FLIPI scores between FL cases and controls, verifying no composite clinical gradient conferring bias between the groups (*P* = 0.12). There was no significant OS difference from time of low‐grade diagnosis between the transformed and control cohorts (Figure 1, *P* = 0.43).

### 3.2 Measurement of PET metrics

**FIGURE 2:**
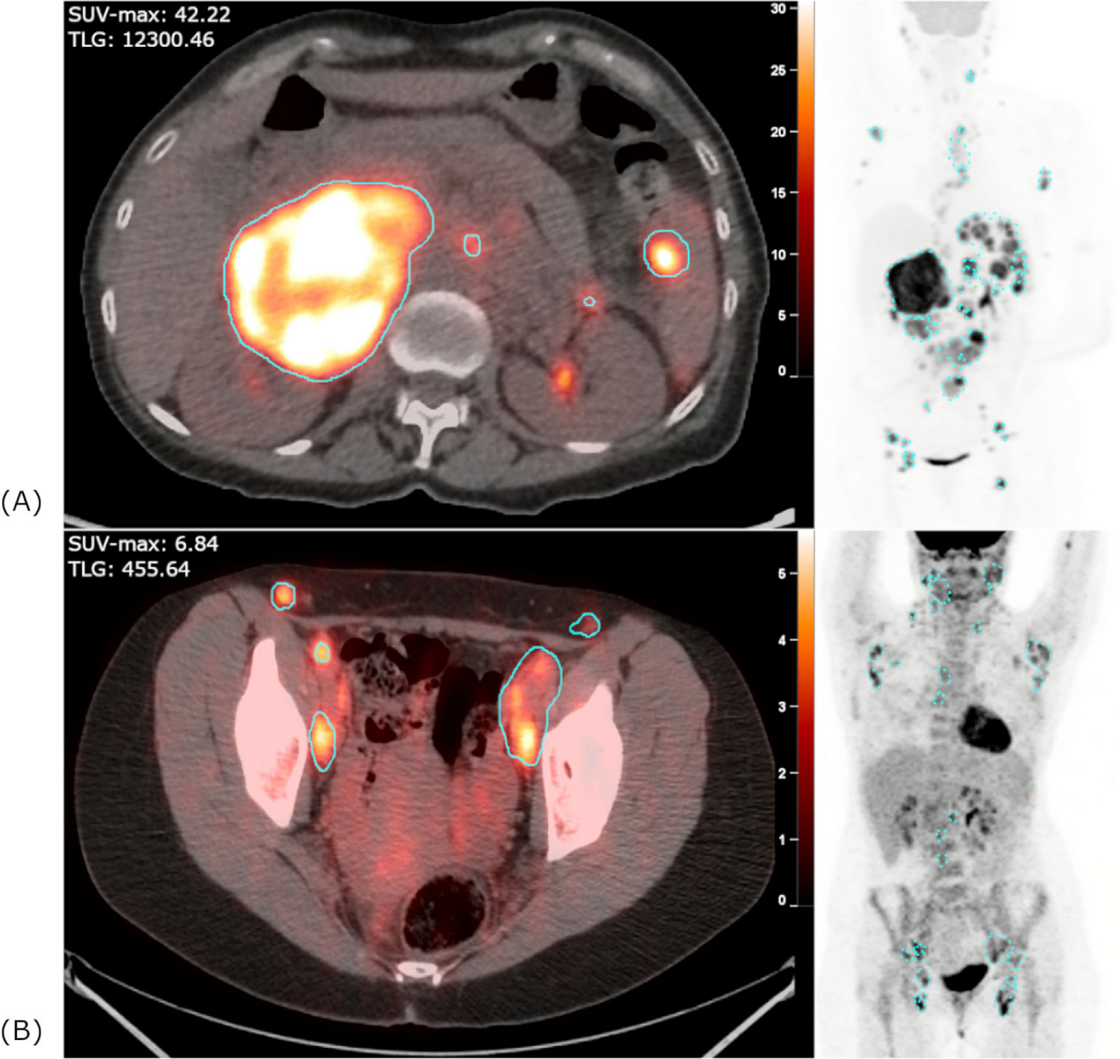
**Representative example of a patient who experienced large cell transformation with high maximum standard uptake value (SUV_max_**) **and total lesion glycolysis (TLG, panel A) versus a control patient (panel B) with lower SUV_max_ and TLG**. For both cases, a representative axial positron emission tomography (PET)/computed tomography (CT) slice and the corresponding maximum intensity projection (MIP) reveal the more intense SUV_max_ and higher volume disease burden for the transformed case in panel A (SUV scale 0–30) as compared to the control case in panel B (SUV scale 0–5.5).

**FIGURE 3:**
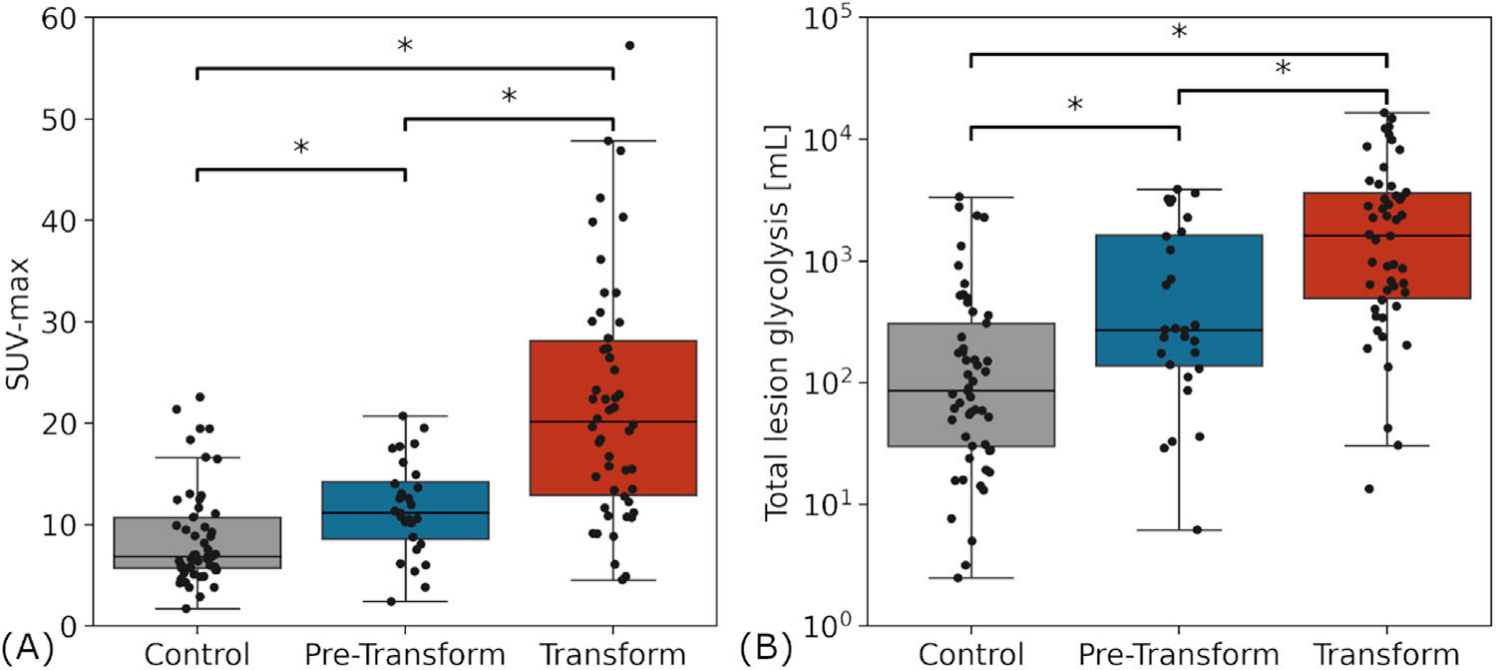
**Comparison of maximum standard uptake value (SUV_max_**) **(panel A) and total lesion glycolysis (TLG, panel B) for patients without transformation (grey) as well as patients with low‐grade lymphoma who later transformed (blue) and patients at the time of high‐grade transformation (red)**. Scattered boxplots demonstrate patients who experienced transformation have significantly higher SUV_max_ at initial diagnosis of low‐grade lymphoma as well as at diagnosis of high‐grade lymphoma as compared to a control cohort who did not experience transformation. *P< 0.05 by unpaired Student's *t*‐test

For the 53 cases of transformation, 28 (53%) had a PET prior to transformation with median time difference of 34.3 months (range: 2.1–157.7) between the scan and transformation. Fifty (94%) had a PET at time of transformation, and 25 (47%) had a PET at both time points. For the control cohort, all 53 patients had a PET at time of initial diagnosis. Figure 2 demonstrates a representative example of a case and control with the ROI outlined in blue. MIM PET Edge gradient‐based MTV segmentation did not visually differ substantially from trialed thresholding‐based methods using 41% of the SUV‐max. For the subgroup of cases with a PET prior to transformation, we observed a median ROI number of 8 (range: 1–51), mean SUV‐max was 11.61 (range: 2.40–20.71, Figure 3A), mean TLG was 995.33 cubic centimeters (cc, range: 6.16–3883.23, Figure 3B), mean MTV was 210.75 cc (range: 1.01–935.39). For PET scans at time of transformation, median ROI number was 7.5 (range: 1–45), mean SUV‐max was 22.03 (range: 4.54–57.26, Figure 3A), mean TLG was 3256.18 cc (range: 13.37–16530.56, Figure 3B), mean MTV was 423.34 cc (range: 3.46–3379.34). Within the control cohort, with PET scans at time of diagnosis, median ROI number was 5 (range: 1–53), mean SUV‐max was 8.71 (range: 1.70–22.57, Figure 3A), mean TLG was 368.58 cc (range: 2.48–3367.24, Figure 3B), mean MTV was 92.05 cc (range: 0.97–649.36). As demonstrated in Figure 3A, there was a significant incremental increase in SUV‐max between control, pretransformation, and post‐transformation PET scans. Similarly, as shown in Figure 3B, there was a significant incremental increase in TLG between control, pretransformation, and posttransformation PET scans. The comparisons in Figure 3A,B were significant at *P* < 0.05 by unpaired Student's *t*‐test as some patients did not have pretransformation PET imaging.

Of the 28 patients with a PET prior to transformation and at time of biopsy confirmation of low‐grade lymphoma, 12 (43%) obtained a biopsy of the site with greatest SUV on PET. Of those 16 who did not have a biopsy of the SUV‐max site, the median absolute difference in SUV‐max between the biopsied and global maximum site was 5.55 (range: 0.29–16.00). This represents less than a two‐fold difference (11). This difference, driven by the 16 patients, resulted in the means of biopsied SUV‐max and global SUV‐max to differ significantly across the 28 eligible patients (P = 0.018). The mean time to transformation for the 16 patients who did not have a biopsy of the SUV‐max site was 81 months and not statistically significantly different from that of the entire cohort. Finally, for the 28 cases with PET prior to transformation, we did observe a nonsignificant trend for incrementally higher SUVmax (*P* = 0.703) and TLG (*P* = 0.552) for those that transformed earlier after initial diagnosis (<2 years, 2–5 years, and >5 years, Figure S1A,B).

### 3.3 Identification of factors associated with transformation and predictive modeling

**FIGURE 4:**
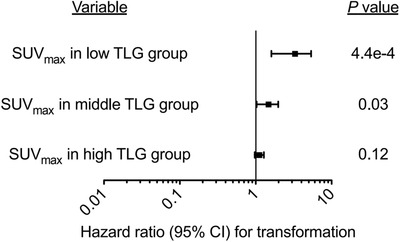
**Forest plot demonstrating the interaction between maximum standard uptake value (SUV_max_**) **and total lesion glycolysis (TLG)**. The forest plot demonstrates the hazard ratio for SUV‐max for the event of transformation by binning the sample of cases and controls into three tiers: low, middle, and high TLG. As shown, the hazard ratio for SUV‐max has the largest magnitude within the low TLG subgroup suggesting SUV‐max may have greater utility in identifying transformed disease for patients with low volume lymphoma.

We performed univariable and multivariable regression analyses (MVA) adjusted for the competing risk of death to identify factors associated with transformation (Table S1). Age, gender, and advanced stage at initial diagnosis of low‐grade lymphoma were not associated with transformation. LDH elevation was associated with transformation at time of high‐grade lymphoma diagnosis but not at the initial low grade time point. All PET metrics (SUV‐max, MTV, TLG) were associated with transformation on univariable analysis (*P* < 0.05). As MTV and TLG were highly collinear (see below regarding correlation matrix), we chose TLG for inclusion in our MVA. We performed an MVA including PET metrics at time of low‐grade lymphoma and observed TLG was the only factor that retained significant association with transformation. Including PET metrics at the time of transformation as well as the control data, we observed SUV‐max and TLG were significantly associated with transformation by MVA. However, there was a significant interaction between SUV‐max and TLG (*P* = 0.04), and as shown in Figure 4 and in Table S1, SUV‐max appeared to have the largest effect size when TLG was smallest.

**FIGURE 5:**
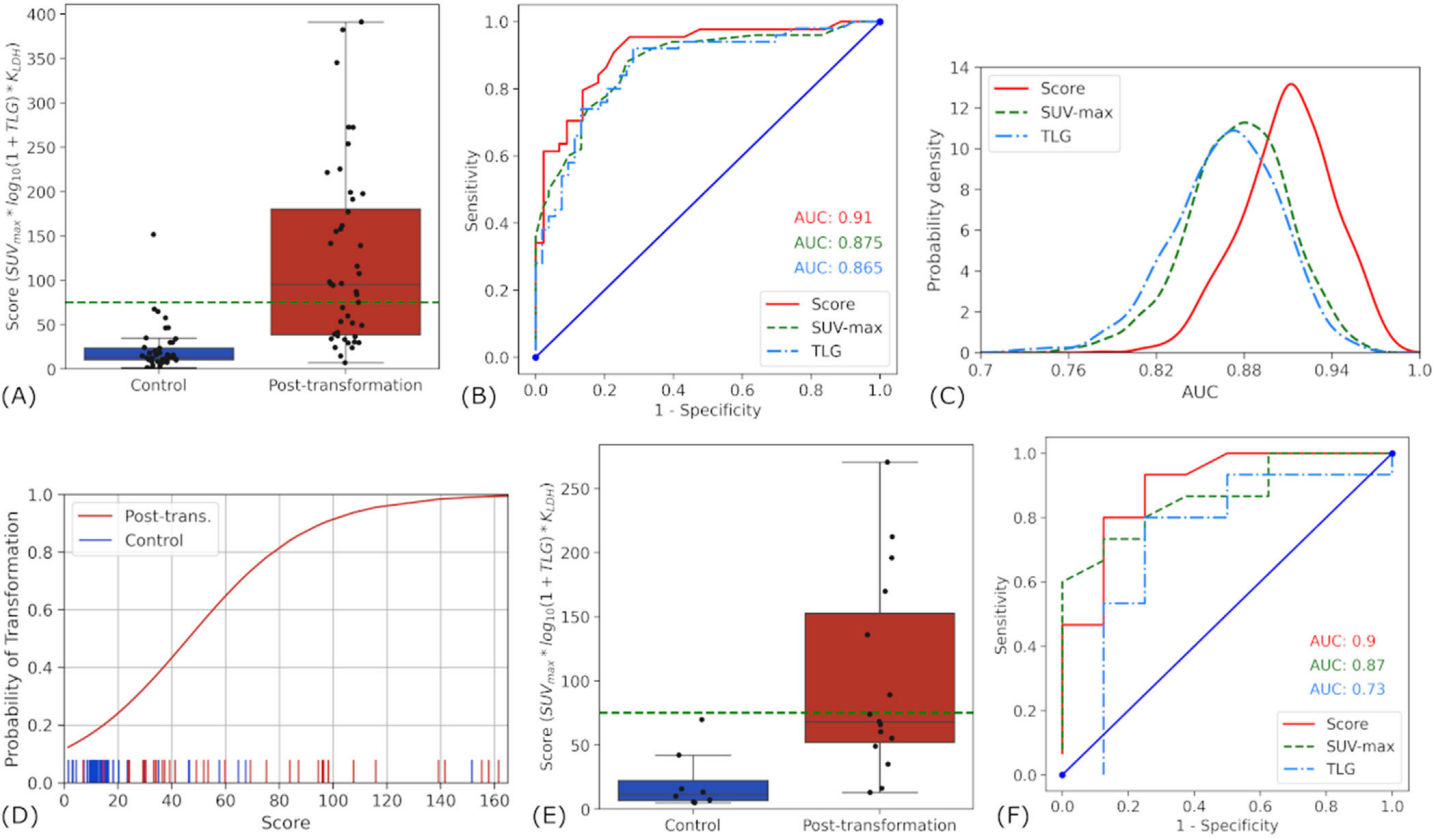
**Evaluation of scoring model to identify transformation status**. (A) Boxplots of model score values across control versus posttransformation groups, with green horizontal line at a score of 75 units representing recommended threshold. (B) **Receiver operator characteristic (ROC) curve and area (AUC)** of various threshold values of the model score versus maximum standard uptake value (SUV_max_) and total lesion glycolysis (TLG) in predicting transformations. (C) **Histogram density distributions** of the 1000 bootstrap iteration AUC results for each predictor, with significant improvement in the model over the SUV‐max or TLG alone. (D) **Logistic regression and associated rug plot** demonstrating an estimated probability of transformation as a function of the model score generated from the discovery cohort. Rug plot ticks exist beyond the displayed domain; however, the x‐axis is restricted to optimize display. (E) **Boxplots** of model score values across control versus posttransformation groups in the validation cohort demonstrating similar behavior to the primary cohort with the same recommended threshold of 75 units. (F) **ROC curve and area (AUC)** of various threshold values of the model score versus SUV‐max and TLG in predicting transformations within the validation cohort portraying similar trends to the primary cohort with a score AUC value of 0.90

We next sought to develop and examine a predictive scoring system reflecting the nested and collinear nature of the PET metrics as well as other prognostic factors (see methods). We measured a model score of 50.8 (range: 3.6–349.0) for controls, 62.1 (range: 8.2–130.6) for cases at pretransformation, and 282.8 (range: 16.2–900.6) for cases at time of transformation as shown in Figure 5.

The justification behind the model formula is to account for the contribution of three key factors in determining transformation. Two continuous and nested PET factors are the SUV‐max and TLG, while a categorical LDH elevation acts as a binary scalar. Although SUV‐max will typically correlate with TLG, the two are relatively separate in classifying two groups of transformed lymphomas (as discussed above and shown in Figure 4). The first group has low‐burden, high‐avidity disease, while the second has high‐burden but lower‐avidity disease. A multiplicative relationship between the two allows for both states to be represented. The base‐10 logarithm operating on the TLG is to limit the marginal predictive utility of ever‐increasing disease volume. It also aids to produce results in similar order of magnitude to the SUV‐max. Finally, the nonradiologic LDH scalar serves as a serum biomarker of the aggressiveness of the lymphoma, which cannot be obtained solely via PET. We also evaluated a model which incorporated the linear additive terms of SUV‐max, TLG and LDH elevation with concern for the Principle of Marginality, but that model had a lower area under the curve (AUC) of 0.883 and as the terms SUV‐max and TLG are nested (see methods) we selected the final model based on optimizing performance.

Our model was successful in clustering the non‐transformed PET studies (control and pretransformed scans) by score values and separating them from transformed studies. There was no SS difference in the means of the control score and pretransformation score of 22.06 versus 26.98 (*P* = 0.422). However, there was a significant difference between the model scores of the controls and posttransformation cases (*P* < 0.001, Figure 5A). In further characterizing the model score in distinguishing between control and posttransformed PET features we measured an AUC of 0.91 (Figure 5B). This was internally validated using 1000 iterations of a bootstrap cross‐validation re‐sampling, yielding a mean AUC of 0.910 (95% CI: 0.847–0.965). This was significantly greater than both SUV‐max and TLG independently (*P* < 0.001) (Figure 5C). To optimize specificity, a model score threshold of 75 was selected. This yielded a sensitivity of 59% and an impactful specificity of 98% in the detection of a posttransformed lymphoma. Of note, given the mixture of histologies in the case and control groups, we also measured our model performance including FL only and observed very similar results (Figure S2). Lastly, we generated a logistic probability of transformation using the model score (Figure 5D).

### 3.4 Validation of results

A total of 23 patients were analyzed as part of a validation cohort, which included eight patients without transformation and 15 patients who experienced transformation. The majority of patients had a low‐grade diagnosis of FL (seven of eight and 13 of 15 respectively). The control group had a mean SUV‐max of 8.12 (range: 2.50–17.09), mean TLG of 1664 (range: 18–12062), and mean model score of 21.08 (range: 4.95–69.75). The transformation group had a mean SUV‐max of 21.15 (range: 5.57–44.14), mean TLG of 2766 (range: 13–11880), and a mean model score of 100.68 (range: 13.01–270.39). We validated that the model score was significantly different between the two group (*P* = 0.011).

The validation groups demonstrated appropriate segregation with our score threshold of 75 determined in our primary study cohort (Figure 5E). This threshold maintained a high specificity of 100%. ROC analysis corroborated our prior findings with greatest AUC of 0.90 utilizing the model score, compared to SUV‐max or TLG alone (0.87 or 0.73) (Figure 5F).

## 4 DISCUSSION

In this study, we investigated the utility of PET metrics in identification of transformed lymphoma and subsequently developed a predictive scoring system. To our knowledge, this is the first case‐control study focusing on multiple PET features of low‐grade lymphoma transformation and developing a model scoring systemic for differentiating the histological aggressiveness of lymphoma present. The majority of our patients had FL and advanced stage at time of initial diagnosis of low‐grade lymphoma. We successfully identified a control cohort with similar baseline clinical characteristics, follow‐up time compared to time to transformation, and histology. The median time to transformation was between 8 and 9 years after initial low‐grade diagnosis, and occurred with a mode between ages of 60–70 years. The most common histology to transform was FL, likely reflecting its higher incidence among non‐Hodgkin lymphomas. Almost 90% of transformations were to DLBCL. Our primary objective was to determine if PET metrics could distinguish high‐grade from low‐grade lymphoma but also to examine the predictive utility of employing the imaging‐based biomarkers.

Looking at our imaging analyses, we found that transformation cases had a higher SUV‐max and TLG both prior to and at the time of transformation as compared to the control group. This is similar to results reported by others [10, 11, 19, 20]. Noy et al. previously reported >80% and >90% specificity in identifying high grade lymphoma for cases with SUV‐max >10 and >13, respectively [10, 21]. Karam et al. showed that patients without transformation had similar SUV‐max values on serial PET imaging and that transformation was unlikely for patients with sites other than the global SUV‐max site biopsied [11]. The GALLIUM study investigators did not observe association between SUV‐max and transformation [12]. However, as the median follow‐up from that study was only 59 months, further follow‐up with additional events and possibly inclusion of other PET parameters such as MTV or TLG may identify a significant association between PET metrics and histologic transformation. Additionally, nearly all patients enrolled on GALLIUM had advanced stage FL with ∼40% having high risk FLIPI scores, which contrasts the baseline factors for patients in our discovery cohort. While we observed an increase in SUV‐max when comparing transformed cases versus controls, our study is unique in characterizing the multiplicative interaction between SUV‐max and TLG, suggesting SUV‐max may substitute as a better indicator of high‐grade disease for patients with low volume lymphoma.

Finally, combining our findings mathematically, we developed a model incorporating SUV‐max, TLG, and LDH that has high predictive ability to identify transformed lymphoma for our entire patient cohort as well as for the FL‐only subset (AUC ≥ 0.9). Our model was generated as a result of multivariable regression analyses. Our independent second cohort provided validation that our model scoring system may allow for a highly specific imaging‐based biomarker indicative of high‐grade transformations. This may translate to cases where pathological confirmation of large‐cell transformation cannot be obtained but clinical behavior of the neoplasm suggests high‐grade disease. Such situations may arise from either contraindication to certain biopsy modalities or from sampling errors not capturing potentially present large‐cell components.

We acknowledge the presence of limitations in our study, which may influence its generalizability or accuracy in all cases. Foremost, the retrospective design of the study harbors intrinsic susceptibility to selection and statistical biases and uncontrolled confounding factors. Similarly, practice patterns, including PET technique changed over the extended period of study. We also included a heterogenous mix of low‐grade histologies. Given the time‐dependent criteria of large‐cell transformation, there is a risk of control patients not yet having transformed but would in the future. We attempted to mitigate the impact of these factors via histological matching to the most common low‐grade lymphoma (FL) as well as to the time‐to‐transformation with control follow‐up times. This resulted in the follow‐up times for cases being significantly longer than that of controls. A variable not matched for was staging with a nonsignificant numerically higher incidence of advanced stage in the case cohort. Given the large percent of patients without biopsy of the most FDG avid site among pretransformation cases (57%), there is a risk of un‐sampled large‐cell transformations. However, as transformations occurred for these patients with biopsies of non‐SUV‐max sites along a temporal distribution not different than the overall cohort, it would appear their disease was not behaving as already transformed. Further, pretransformation PET scans were not universally acquired at initial time of diagnosis, although the scans available for our study were accompanied by pathological verification of low‐grade lymphoma. The model score is based on two PET metrics, which have some correlation, as well as influence from stage, and thus may provide redundant information in radiological classification. Finally, despite successful validation of our primary findings in an independent cohort, we acknowledge this second group is limited in numbers without equal representation of cases and controls, as was present with our discovery cohort.

Overall, our study demonstrates a radiological association of transformed lymphomas with higher SUV‐max and TLG. Our model's scoring system employs this to significantly provide noninvasive stratification between the two states with a high degree of specificity. Future investigations are required to externally and prospectively validate this model with pathologic evidence of transformation.

## AUTHOR CONTRIBUTIONS

All authors reviewed and assisted with writing of the manuscript. Furthermore, J.O. analyzed data, S.M.H. contributed to the patient cohorts, J.S.M. performed research, H.H.G. contributed analytical tools, H.J.N. contributed to the patient cohorts, E.J.M. contributed to the patient cohorts, R.H.A. contributed to the patient cohorts, A.A.A. contributed to the patient cohorts and designed research, R.T.H. contributed to the patient cohort, M.S.B. designed research and analyzed data.

## CONFLICT OF INTEREST

The authors declare that there is no conflict of interest that could be perceived as prejudicing the impartiality of the research reported.

## FUNDING INFORMATION

There was no funding source for this study.

## ETHICS STATEMENT

The authors performed a retrospective case‐control study of patients seen at the Stanford Cancer Institute from 2003 through 2020 with institutional board review (IRB) approval. Due to the retrospective nature, patient consent was not required by our ethics board. This was an IRB approved retrospective study with omission of patient consent due to the retrospective nature.

## Supporting information

Supporting Information

## Data Availability

Patient data are not available for upload due to HIPAA protocols. All non‐HIPAA data are included in summary tables.
