# Supplementary material for: Investigating and modeling positron emission tomography factors associated with large cell transformation from low‐grade lymphomas
Source: EJHaem. 2022 Nov 25;4(1):90–9. doi: 10.1002/jha2.615 (PMC9928791; doi:10.1002/jha2.615)

Supplemental Table 1. Results of univariable and multivariable (MVA) regressions associated with transformation, revealing SS findings in SUV-max, MTV, TLG, LDH elevation and a product of SUV-max and TLG (logarithmic).

Supplemental Table 1. Univariable and multivariable analyses for transformation.

Variable		Univariable	MVA1 (28 events)	MVA2 (51 events)
Age	HR<br>95% CI<br>*P*	1.01<br>0.99-1.03<br>0.28		
Male gender	HR<br>95% CI<br>*P*	1.34<br>0.77-2.35<br>0.30		
Stage III-IV	HR<br>95% CI<br>*P*	1.63<br>0.9-2.95<br>0.11		
SUVmax, pretransformation (per 5 unit increase)	HR<br>95% CI<br>*P*	**1.45**<br>**1.02-2.06**<br>**0.039**	1.20<br>0.78-1.83<br>0.41	
Log_{10}(MTV), pretransformation	HR<br>95% CI<br>*P*	**1.79**<br>**1.05-3.03**<br>**0.031**		
Log_{10}(TLG) pretransformation	HR<br>95% CI<br>*P*	**2.02**<br>**1.25-3.26**<br>**0.004**	**1.78**<br>**1.02-3.14**<br>**0.044**	
SUVmax, post-transformation (per 5 unit increase	HR<br>95% CI<br>*P*	**1.22**<br>**1.13-1.33**<br>**1.6e-6**		**2.09**<br>**1.01-4.35**<br>**0.048**
Log_{10}(MTV), post-transformation	HR<br>95% CI<br>*P*	**2.24**<br>**1.46-3.43**<br>**0.0002**		
Log_{10}(TLG), post-transformation	HR<br>95% CI<br>*P*	**2.16**<br>**1.55-2.99**<br>**4.1e-6**		**2.98**<br>**1.34-6.64**<br>**0.008**
SUVmax*Log_{10}(TLG), post-transformation	HR<br>95% CI<br>*P*			**0.96**<br>**0.92-0.99**<br>**0.042**
LDH elevation, pretransformation	HR<br>95% CI<br>*P*	0.61<br>0.11-3.47<br>0.58		
LDH elevation, post-transformation	HR<br>95% CI<br>*P*	**2.43**<br>**1.38-4.30**<br>**0.002**		1.48<br>0.72-3.05<br>0.29

Abbreviations: MVA=Multivariable analysis; MTV=metabolic tumor volume; TLG=total lesion glycolysis

Supplemental Figure 1. Boxplots of pre-transformation SUV-max (A) and TLG (B) distributed along categorical bins of time-to-transformation. ANOVA yielded no difference between the values across the < 2 years, 2-5 years and > 5 years groups.

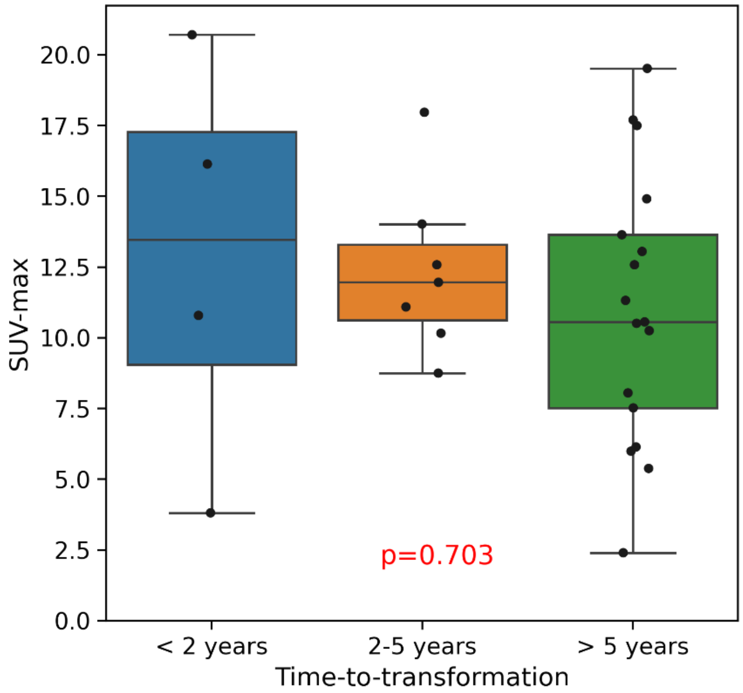

(A)

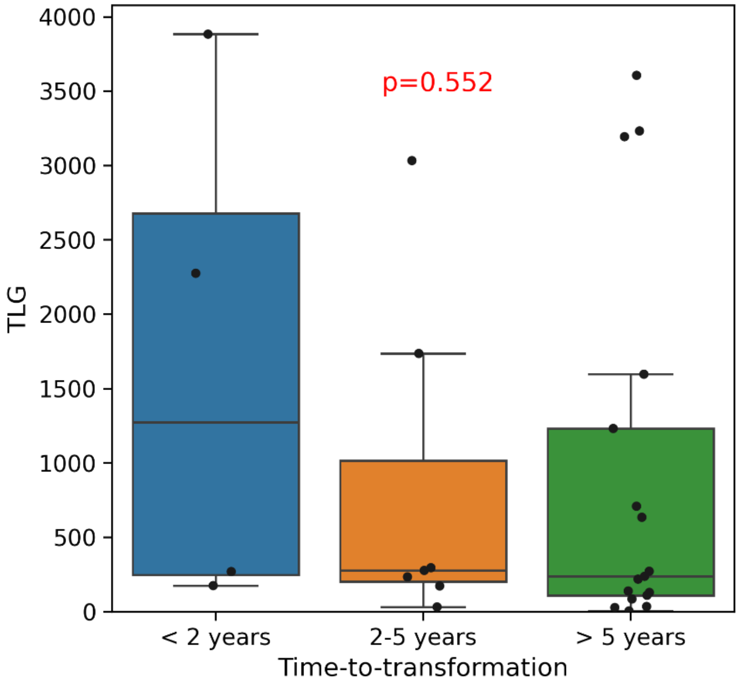

(B)

Supplemental Figure 2. ROC curve and area (AUC) within follicular lymphoma only patients of various threshold values of the model score vs SUV-max and TLG in predicting transformations. These curves resemble those displayed in the entire cohort, exhibiting consistency in our findings.

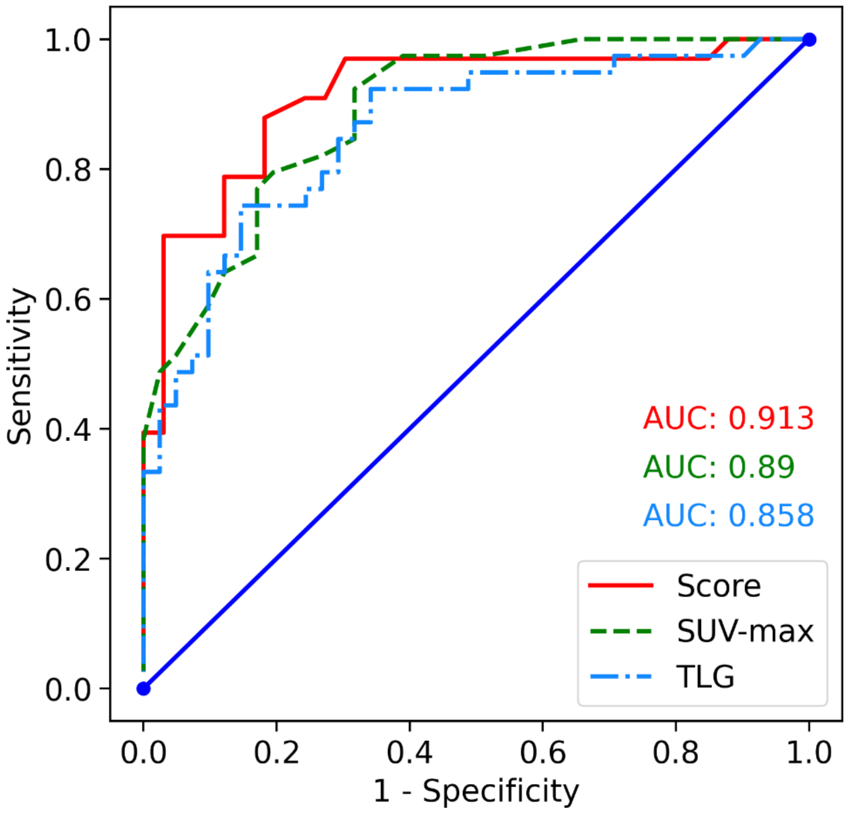

Supplement: Supplementary file 1 — Supporting Information [file JHA2-4-90-s001.pdf]
